# Geographic disparities in physical and mental health comorbidities and socioeconomic status of residence among Medicaid beneficiaries in Utah

**DOI:** 10.3389/fpubh.2024.1454783

**Published:** 2025-01-06

**Authors:** Roberta Z. Horth, Srimoyee Bose, Carl Grafe, Navina Forsythe, Angela Dunn

**Affiliations:** ^1^Epidemic Intelligence Service, Division of Scientific Education and Professional Development, Centers for Disease Control and Prevention, Atlanta, GA, United States; ^2^United States Public Health Service, Rockville, MD, United States; ^3^Utah Department of Health, Salt Lake City, UT, United States; ^4^CDC Steven M. Teutsch Prevention Effectiveness Fellowship, Division of Scientific Education and Professional Development, Centers for Disease Control and Prevention, Atlanta, GA, United States; ^5^Public Health Informatics Fellowship, Division of Scientific Education and Professional Development, Centers for Disease Control and Prevention, Atlanta, GA, United States

**Keywords:** physical health, mental health, Medicaid, GIS, social determinants, comorbidity

## Abstract

To examine the relationship between socioeconomic deprivation and complex needs, defined as mental and physical comorbidities, we conducted a cross-sectional retrospective cohort analysis of adult Utah Medicaid beneficiaries. Our analysis included Medicaid beneficiaries with geocoded addresses aged ≥18 years in Utah (*N* = 157,739). We geocoded beneficiary addresses and assigned them to census block groups. We compared the socioeconomic status of block groups (Singh’s area deprivation index) with the proportion of complex needs, defined based on cluster analysis as 1 physical condition with depression or ≥ 2 physical with ≥1 mental health condition. Spatial mapping was performed of prevalence quantiles grouped by count overlaid with Medicaid-covered mental health facilities. Prevalence of complex needs was 18.9% (*n* = 29,742); beneficiaries with >3 emergency department visits had 12.8 odds of having complex needs; 39.7% of beneficiaries with >$5,000 in annual costs had complex needs. Common comorbid conditions among beneficiaries with complex needs were hypertension (56.0%), hyperlipidemia (35.5%), depression (68.8%), anxiety (56.2%), drug use (16.0%), and alcohol use disorders (15.2%). Census block groups with higher deprivation had a higher proportion of complex needs (*ρ* = 0.21, *p* < 0.001). There was a statistically significant spatial autocorrelation of the prevalence of complex needs (Moran’s I index: 0.65; *p* < 0.001). Six high-count census blocks had no mental health facilities. Areas with increased socioeconomic deprivation had a greater proportion of complex needs and fewer mental health facilities. Integrated programs addressing both physical and mental health conditions with a focus on socioeconomically deprived areas might benefit Medicaid recipients in populations such as those in Utah.

## Highlights

- One in five Medicaid beneficiaries had mental and physical comorbidities (complex needs).
- Increased socioeconomic deprivation score is associated with increased complex needs.
- There is spatial autocorrelation of the prevalence of complex needs.
- Areas with greater deprivation need more integrated mental and physical health services.

## Introduction

Beginning in late 2015, Utah Medicaid identified a limited proportion of substantial need and substantial healthcare use patients who accounted for a disproportionate expenditure of Medicaid costs. Some beneficiaries in the top percentile of healthcare costs have frequent contact with high-cost healthcare services that may not be fully addressing their complex needs, particularly those needs related to comorbid physical and mental health conditions (1). Typically, persons with physical and mental health comorbidities have poorer health outcomes and higher healthcare costs (2). Medicaid beneficiaries with physical and mental health comorbidities cost up to 75% more than those without mental health comorbidities (3).

The high proportion of mental health conditions among Medicaid beneficiaries with physical health conditions nationally has been documented (2). Despite the presence of high levels of physical and mental health comorbidities among beneficiaries, Medicaid-covered mental health services have historically been segregated from physical health services (4). This fragmentation of the healthcare system makes both mental health and physical health services more difficult to access, resulting in unmet patient needs and increased healthcare costs (5).

Physical and mental health comorbidities are exacerbated by socioeconomic deprivation (6, 7). A better understanding of the healthcare needs of these patients and the socioeconomic factors that affect healthcare use can help achieve healthcare cost containment and improve population health outcomes (8).

Limited information is available regarding the association between comorbid physical and mental health conditions and geographical socioeconomic disparities among Utah Medicaid beneficiaries. To fill this knowledge gap, we sought to characterize the complex needs of Utah Medicaid beneficiaries and examine how complex needs are associated with socioeconomic deprivation as measured by the Singh area deprivation index (ADI) (9). We hypothesized that socioeconomic deprivation was associated with an increased prevalence of comorbidities.

## Methods

### Study design and eligibility criteria

We conducted a cross-sectional retrospective cohort analysis of Utah Medicaid claims data from the Division of Medicaid and Health Financing at the Utah Department of Health (UDOH). Medicaid claims data are the medical bills submitted to Utah Medicaid for the services provided to Medicaid beneficiaries by care providers; each claim is associated with a date of service. To capture the entire population of Medicaid beneficiaries in a 12-month period, we reviewed claims data for all Medicaid beneficiaries eligible for at least 1 month in Utah during Fiscal Year 2017 (from 1 July 2016 to 30 June 2017). We extracted Utah Medicaid claims costs using Structured Query Language from the Medicaid centralized data warehouse, which contains patient-managed care, fee for service, and pharmacy claims. Claims costs during the 12 months before a beneficiary’s most recent claim based on the date of service in the study period were used. This design was selected in order to simulate rolling patient identification, which is more representative of data that would be used operationally to identify high utilizers and less likely to be biased by an arbitrary time cut point that might not capture changes in care-seeking behavior (10). Persons eligible for at least 1 month during the 12 months without any medical claims were assigned a cost of $0. Data were collected at the individual patient level and included the patient’s unique identifier, date of birth, number of months they were eligible for Medicaid, diagnosis codes, address, demographic information, and medical costs.

The sampling population included beneficiaries enrolled in managed care or fee-for-service. Refugees and custody medical patients were excluded. We limited the analysis to adults aged ≥18 years. The Institutional Review Boards at the Utah Department of Health (Approval #533) and the Centers for Disease Control and Prevention (HSR #2018-00189) determined that this study did not constitute human subjects’ research.

### Data analysis

The *International Classification of Diseases*, Tenth Revision and the *International Classification of Diseases*, Ninth Revision, Clinical Modification (ICD-10-CM and ICD-9-CM) codes from claims data were used to define the main outcome of interest: beneficiaries with chronic physical and mental health conditions (11, 12). Chronic disease algorithms from the Centers for Medicare & Medicaid Services Chronic Conditions Data Warehouse (CCW) were used to classify chronic disease combinations (13). Based on previous published analysis conducted on the data, which identified that those with complex physical and mental health conditions were clustered (14), we defined complex needs of Medicaid beneficiaries as a chronic physical condition plus a mental health condition from the CCW Chronic Conditions categories (CCW-CC), or at least two physical conditions and any mental health condition from the CCW Other Chronic, Mental Health, or Potentially Disabling Conditions categories (CCW-OC) (Supplementary Figure 1).

Beneficiary addresses were geocoded using ArcGIS 10.5 (Environmental Systems Research Institute, Redlands, CA, USA). Software matching option parameters were set to 70% for spelling sensitivity, 70% minimum candidate score, and 70% minimum score match. A comparison of matched to unmatched addresses revealed that more addresses in urban areas vs. those in rural areas were geocoded. Because of this, we assigned a population-adjusted centroid value to any address having a zip code comprising a single census block group (all rural areas in Utah) with >50% unmatched addresses. Persons with missing addresses and those whose address was listed as homeless were excluded from the geocoding process. All geocoded addresses were assigned to their census block groups.

Because Utah’s Medicaid data lack information on key socioeconomic characteristics of beneficiaries, we used the 2015 Singh area deprivation index (ADI) to look for area-level effects of socioeconomic position. The ADI calculates a score by census block group using 17 U.S Census Bureau measures that characterize the level of socioeconomic need in a neighborhood (9). Higher index values represent higher levels of deprivation. We applied Utah-specific ADI quintiles adapted from Knighton et al. (15) from the original Singh method. Knighton et al. quintiles are the rounded census block group area deprivation scores binned into five approximately equal size groups, from lowest score (least deprivation) to highest score (highest deprivation). We calculated the proportion of beneficiaries with complex needs and ADI quintiles at the census block group level. We calculated Spearman’s rank correlation to measure the association between these two variables.

We measured healthcare utilization by the number of emergency department visits, number of hospital inpatient visits, and dollar amount in claims, including for managed care patients, during the study period. We used the X^2^ test to assess trends in the proportion of beneficiaries with complex needs by utilization. We used mixed-effects multivariable logistic regression (*glmr* in the *lme4* package in R v3.5.3 [R Foundation for Statistical Computing, Vienna, Austria]) including census block group as a random effect to measure the association of complex needs with area deprivation index quintile and healthcare utilization while adjusting for sex, age groups (categorized as 18–40, 41–65, and > 65), ethnicity, and number of months (categorized as 1–3, 4–6, 7–9, and > 9) on Medicaid. We tested for multicollinearity using the variance inflation factor. We conducted an analysis of variance to compare full and reduced models. We conducted a sensitivity analysis excluding beneficiaries with no claims and those with <12 months of continued eligibility for Medicaid. We present odds ratios and 95% confidence intervals for the full model, which demonstrated the best fit.

We used GeoDa (16) and QGIS (17) software for spatial mapping of counts and proportions of complex needs. The census block group-level map was overlaid with Medicaid-covered mental health facility locations to find the census blocks with the highest counts and proportions of beneficiaries with complex needs. We also identified the census block groups with the highest counts and limited or no access to mental health facilities. Local Empirical Bayesian Moran’s I statistic (16) measured the presence of spatial dependence of the proportions of complex needs at the census block level in Utah.

## Results

### Descriptive statistics

Among adult beneficiaries, 157,739 (93.9%) beneficiary addresses were geocoded. The proportions with complex needs were similar between Medicaid beneficiaries whose addresses had been geocoded and those whose addresses had not (18.9% geocoded vs. 18.1% non-geocoded, *p* = 0.07, Supplementary Table 1). Only beneficiaries with geocoded addresses were included in the analysis. Among these, 60.9% were aged 18–40 years, 65.5% were women, and 12.6% were Hispanic. Additionally, 75.5% were enrolled in Medicaid for more than 9 months, 4.3% had visited the emergency room more than three times, and 2.2% had greater than two hospital admissions in 12 months.

Among beneficiaries with complex needs (*n* = 29,742), the most common chronic physical conditions included hypertension (56.0%), rheumatoid arthritis or osteoarthritis (35.5%), hyperlipidemia (33.7%), and diabetes (30.7%). The most common mental health conditions were depression (68.8%), anxiety disorders (56.2%), and traumatic brain injury and non-psychotic mental disorders attributed to brain damage (24.8%) (Table 1). Among beneficiaries with complex needs, the prevalence of drug use disorders and alcohol use disorders was 16.0 and 15.2%, respectively. The most common comorbidities were hypertension and depression (35.8%), hypertension and anxiety disorders (30.5%), and rheumatoid arthritis or osteoarthritis and depression (24.5%).

**Table 1:** Common comorbidities among Medicaid adult beneficiaries with complex needs (physical and mental health comorbidities) (*n* = 29,742), Utah – 2017.

		Comorbidities with the most common mental health conditions					
		Depression	Anxiety disorders	TBI	Drug use disorders	Bipolar disorder	Alcohol use disorders
Physical conditions	No. (%)	20,452 (68.8)	16,715 (56.2)	7,384 (24.8)	4,758 (16.0)	5,066 (17.0)	4,532 (15.2)
Hypertension	16,664 (56.0)	10,642 (35.8)	9,082 (30.5)	4,406 (14.8)	2,628 (8.8)	2,588 (8.7)	2,710 (9.1)
Rheumatoid arthritis or osteoarthritis	10,570 (35.5)	7,282 (24.5)	6,349 (21.3)	2,960 (10.0)	2,075 (7.0)	1,827 (6.1)	1,662 (5.6)
Hyperlipidemia	10,033 (33.7)	6,325 (21.3)	5,263 (17.7)	2,633 (8.9)	1,234 (4.1)	1,528 (5.1)	1,470 (4.9)
Diabetes	9,126 (30.7)	5,715 (19.2)	4,527 (15.2)	2,555 (8.6)	1,204 (4.0)	1,373 (4.6)	1,281 (4.3)
Chronic kidney disease	8,058 (27.1)	5,056 (17.0)	4,203 (14.1)	2,427 (8.2)	1,438 (4.8)	1,237 (4.2)	1,374 (4.6)
Asthma	6,980 (23.5)	5,090 (17.1)	4,531 (15.2)	1,976 (6.6)	1,337 (4.5)	1,527 (5.1)	1,081 (3.6)

TBI, traumatic brain injury.

### Bivariable and multivariable analyses

Beneficiaries with complex needs differed from those without complex needs in their ADI quintile, number of emergency department visits, number of hospital inpatient visits, and dollar amount paid in claims (Table 2). In our multivariable mixed-effects logistic regression that controlled for sex, age, ethnicity, and number of months on Medicaid, the proportion of beneficiaries with physical and mental health comorbidity increased significantly (*p* < 0.001) with deprivation quintile, reaching 20.4% among beneficiaries in the most deprived quintile compared to 15.2% among beneficiaries in the least deprived quintile. The odds of having complex needs among beneficiaries with ≥3 emergency department visits were greater than for beneficiaries with no visits (12.80, 95% confidence interval [CI] 12.07, 13.56). Similarly, the odds of having complex needs increased with increasing in-patient hospitalizations. The median amount paid in claims for beneficiaries with complex needs was $4,218 while for those without complex needs, it was $1,127. Beneficiaries with >$5,000 in paid claims had 6.26 (95% CI 5.98, 6.55) greater odds of having complex needs compared to patients with $1–$500 in paid claims.

**Table 2:** Medicaid adult beneficiaries (*N* = 157,739) with and without complex needs (physical and mental health comorbidities) by the area deprivation index and healthcare utilization, Utah – 2017.

	With complex needs* No.	Without No.	% w/complex needs	*p* ^a^	AOR^b^	95% CI
Sample size	29,742	127,997	18.9			
ADI quintile^c^						
1 (least deprived)	2,106	11,720	15.2	<0.001		Ref
2	4,876	21,396	18.6		1.16	1.08, 1.26
3	6,159	26,888	18.6		1.24	1.15, 1.34
4	6,663	29,242	18.6		1.22	1.13, 1.32
5 (most deprived)	9,935	38,747	20.4		1.32	1.23, 1.42
Emergency department visits						
0	15,338	106,050	12.6	<0.001		Ref
1	5,426	12,620	30.1		3.09	2.96, 3.21
2	3,002	4,641	39.3		4.60	4.38, 4.86
3	1,856	2,014	48.0		6.75	6.27, 7.27
>3	4,120	2,672	60.7		12.80	12.07, 13.56
Hospital in-patient visits						
0	20,818	109,402	16.0	<0.001		Ref
1	3,407	14,606	18.9		2.26	2.16, 2.36
2	3,046	3,066	49.8		5.50	5.19, 5.84
>2	2,471	923	72.8		14.33	13.16, 15.60
Amount paid in claims (USD) (median, [IQR])	$4,218 ($1,158, $16,694)	$1,127 ($308, $4,895)				
0	74	43,859	0.2	<0.001	0.01	0.01, 0.02
1–500	3,604	27,977	11.4			Ref
501–1,000	3,038	11,270	21.2		2.06	1.95, 2.18
1,001–5,000	9,117	23,808	27.7		3.36	3.21, 3.52
>5,000	13,909	21,083	39.7		6.26	5.98, 6.55

ADI, area deprivation index; Ref, reference value; USD, United States dollars.

a Χ^2^ test for trend.

b AOR, Adjusted odds ratios from mixed-effects multivariable logistic regression adjusting for sex, age, ethnicity, and months on Medicaid.

c Excludes seven persons (three with complex needs and four without) where ADI could not be assigned.

**Figure 1:**
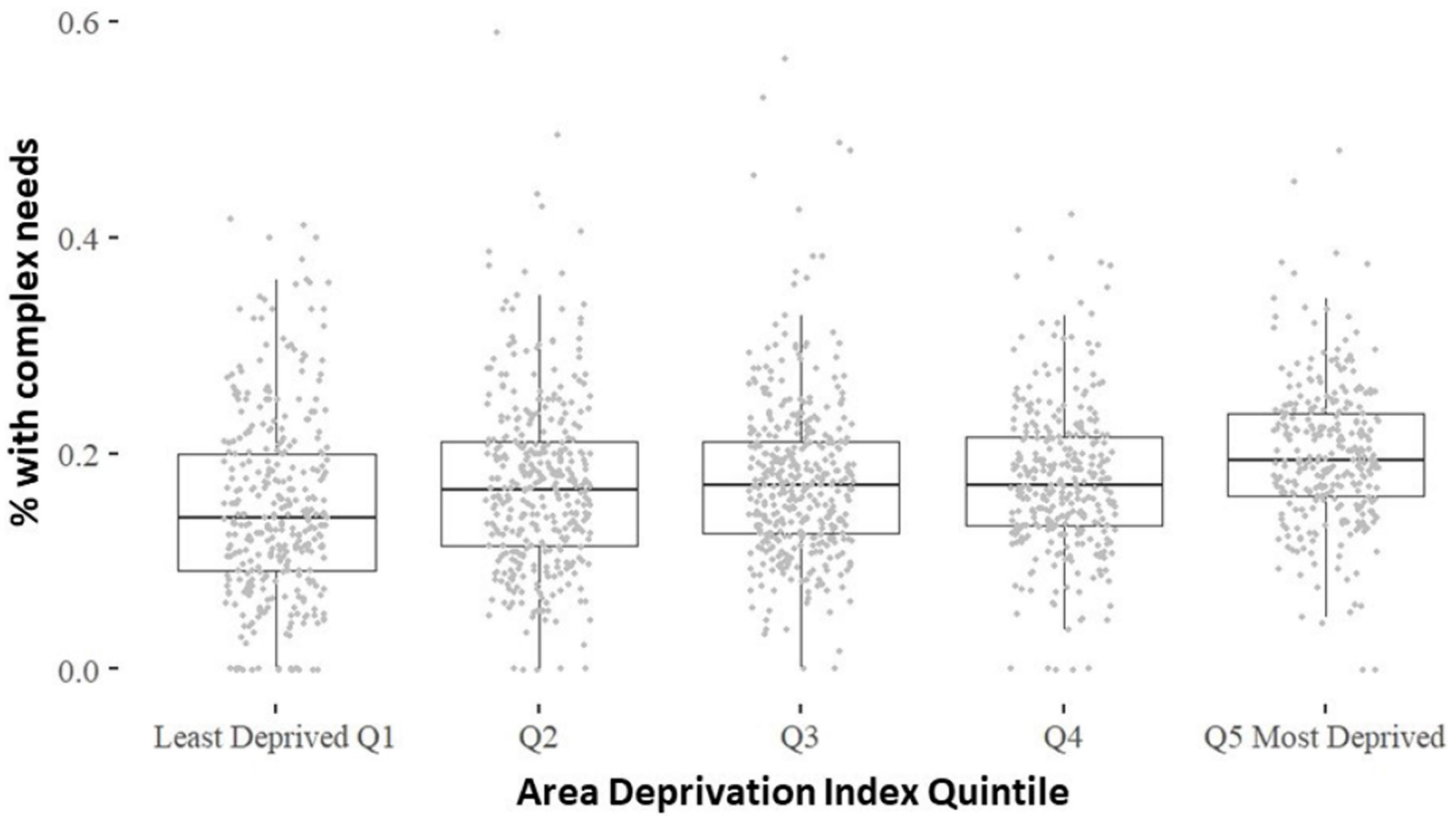
(*p* = 0.21, *p* < 0.001). * 1 physical condition with depression or 22 physical conditions with 21 mental health conditions.

Geocoded adults were assigned to 1,680 census block groups, which were then grouped by their respective ADI quintile. The ADI divides census blocks into five approximately equally-sized groups based on their rounded ADI score, ranging from high to low. Blocks with equal scores were grouped, and each quintile contained from 292 to 377 census block groups. There was a significant positive association between the ADI and the prevalence of complex needs (*ρ* = 0.21, *p* < 0.001) (Figure 1). The median prevalence of complex needs for the lowest ADI quintile was 14.0 and 19.3% for the highest quintile.

### Geospatial analysis

**Figure 2:**
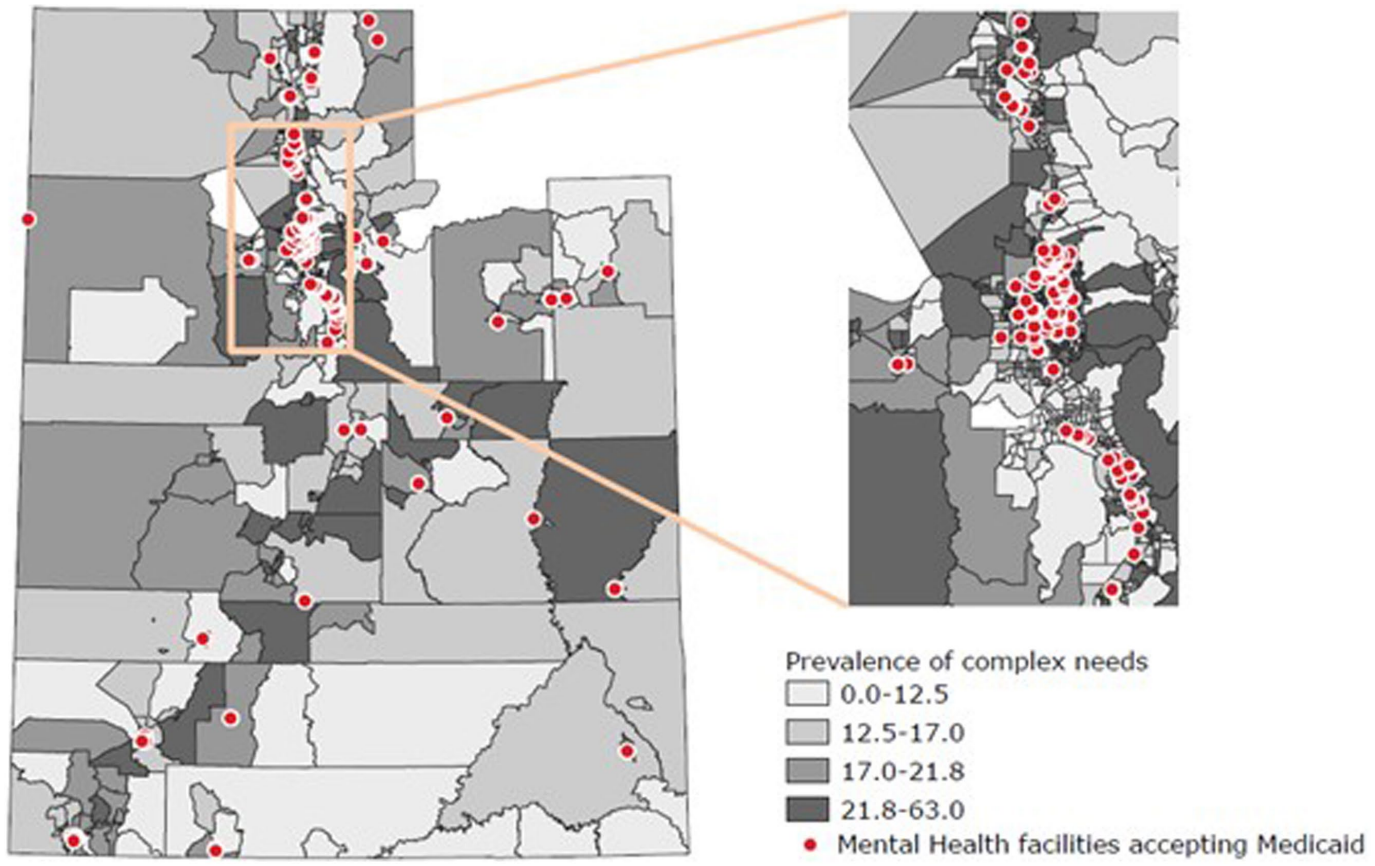
Spatial mapping of proportion of Medicaid adult beneficiaries with complex needs (physical and mental health comorbidities) by census block group and locations of mental health facilities accepting Medicaid — Utah, 2017.

The bivariate spatial mapping of the prevalence of complex needs with the location of Medicaid-covered mental health facilities is presented in Figure 2. There was a statistically significant spatial autocorrelation of the prevalence of complex needs (Moran’s I index: 0.65; *p* < 0.001). This indicates that the high prevalence of beneficiaries with complex needs and the low prevalence of beneficiaries with complex needs are strongly consistent with clustering at the census block level. Among all census block groups, the median number of beneficiaries with complex needs was 12, and the median prevalence was 17%. The highest prevalence quantile (21.8–63.0%) contained 13,304 beneficiaries with complex needs. Within this group, 97 census blocks had the highest counts of beneficiaries with complex needs, totaling 7,250 beneficiaries. The median census block group prevalence of physical and mental health comorbidities in these blocks was 26%. The six census blocks with the highest counts of complex needs had no Medicaid-covered mental health facilities.

## Discussion

In this study, we report that approximately one in five Medicaid adult beneficiaries in Utah had complex needs. This group of beneficiaries contributed disproportionately to the expenditure of resources as measured by the number of emergency department visits, the number of inpatient hospitalizations, and claims costs over the study period. Persons with >3 emergency department visits had approximately 13 times the odds of having complex needs than those with no visits, and persons with >$5,000 paid claims during this 12-month period had six times the odds of having complex needs than those with $1–$500 in paid claims.

Our analysis also demonstrated that at an ecological level, socioeconomic disparities were related to the prevalence of complex needs, with the proportion of beneficiaries with complex needs increasing with increasing socioeconomic deprivation at the census block group level. Our findings are consistent with the literature, which reports that people of low socioeconomic positions and communities with higher deprivation are more likely to be depressed and are more likely to have higher rates of obesity, diabetes, and chronic medical conditions (18). This is likely because beneficiaries living in higher deprivation communities often have greater exposure to stressors that negatively impact health, such as higher rates of pollution, crime, and unemployment, and lower access to services that can improve quality of life, such as safe housing, green spaces, and healthy food options. A United States-wide study of geospatial hotspots found that the highest-ranking census tracts in the nation exhibited poorer mental and physical health. Utah performed better than many other states, with fewer hotspots than neighboring states such as Nevada, New Mexico, and Arizona (19). Geographic conditions associated with disparity have a profound impact on psychological and behavioral factors that influence both physical and mental health (20).

The findings concerning the lack of mental health facilities in locations with high ADI were surprising. Other national-level studies have reported conversely that low-income areas have a higher proportion of outpatient mental health facilities (21). Nevertheless, studies that have reported on mental health providers (instead of facilities) have concluded that similar to our study, communities with higher deprivation have a shortage of mental health providers (22). Expansion of preventive and routine mental health services in underserved areas can result in substantial improvements to mental health and reductions in the use of emergency services and hospitalizations. Additional studies might contribute to an improved understanding of how the availability of mental health facilities in socially deprived regions influences mental and physical health outcomes in Utah.

## Limitations

Our results cannot be generalized beyond the Utah Medicaid population; however, they do provide insight into the association between patients with complex medical needs and the ADI. Inherent bias exists in the use of aggregate characteristics as a proxy for individual deprivation (15). Furthermore, bias is introduced by reliance on healthcare services claims data. Beneficiaries with complex needs who did not have any medical claims in the defined period would have been undercounted, and it is not possible from the data to estimate the extent of this bias. A separate sensitivity analysis was conducted to exclude beneficiaries with no claims or those with <12 months of Medicaid eligibility, revealing no difference in conclusions. We identify the standard limitations of the modifiable area unit problem and the ecological fallacy of the spatial autocorrelation analysis. We also recognize that our analysis was limited to conditions listed in the Medicaid CCW, which is not an exhaustive list of possible chronic physical and mental health conditions. Because address geocoding was not possible for approximately 6.1% of beneficiaries, there might be an underrepresentation of high-deprivation socioeconomic groups that lack a permanent address. However, the prevalence of complex medical needs patients did not differ between beneficiaries with geocoded addresses and those without.

## Conclusion

This study reports on 8% of adults residing in Utah (Utah adult population estimated to be 2,176,739 in 2017) (23). It describes a population that relies on state resources for healthcare needs. Our analysis reveals that areas with greater socioeconomic deprivation have greater comorbid physical and mental health needs and lower access to mental health facilities. In Utah, funding for public mental health services is decentralized, with regions or counties having autonomous local mental health authorities. Literature reveals that decentralization contributes to a lack of integration of physical and mental health services (24). For complex needs patients with mental and chronic physical health comorbidities, integrated healthcare systems might improve health outcomes and reduce use and costs (25). The development of integrated programs addressing both physical and mental health with a focus on socioeconomically deprived areas might benefit Medicaid recipients in populations such as those in Utah. The provision of integrated preventive and clinical care for beneficiaries with physical and mental health conditions might result in reduced high-cost services, such as unnecessary emergent care and inpatient hospitalizations.

## Data Availability

The data analyzed in this study is subject to the following licenses/restrictions: our study used restricted data from Medicaid Utah. Requests to access these datasets should be directed to Utah Department of Health and Human Services, MedicaidOps@utah.gov.
