# Supplementary material for: Geographic disparities in physical and mental health comorbidities and socioeconomic status of residence among Medicaid beneficiaries in Utah
Source: Front Public Health. 2025 Jan 6;12:1454783. doi: 10.3389/fpubh.2024.1454783 (PMC11743258; doi:10.3389/fpubh.2024.1454783)
Supplement: Supplementary file 1 [file Table_1.docx]

**Table 1.** Clinical and Demographic Characteristics of Geocoded and Nongeocoded Medicaid Adult Beneficiaries, Utah — 2017

|  | **Geocoded (n = 157,739)** | **Nongeocoded (n = 10,396)** |  |
| --- | --- | --- | --- |
| **Characteristics** | **No. (%)** | **No. (%)** | ***P*-value** |
| Age (yrs) |  |  |  |
| 18–40 | 96,065 (60.9) | 6,308 (60.7) | <0.001 |
| 41–65 | 43,793 (27.8) | 3,363 (32.3) |  |
| >65 | 17,881 (11.3) | 725 (7.0) |  |
| Sex |  |  |  |
| Male | 54,346 (34.5) | 4,224 (40.6) | <0.001 |
| Female | 103,393 (65.5) | 6,172 (59.4) |  |
| Hispanic ethnicity |  |  |  |
| No | 137,817 (87.4) | 9,352 (90.0) | <0.001 |
| Yes | 19,922 (12.6) | 1,044 (10.0) |  |
| Months enrolled in Medicaid |  |  |  |
| 1–3 | 15,909 (10.1) | 911 (8.8) | <0.001 |
| 4–6 | 11,690 (7.4) | 803 (7.7) |  |
| 7–9 | 11,117 (7.0) | 828 (8.0) |  |
| >9 | 119,023 (75.5) | 7,854 (75.5) |  |
| Emergency department visits |  |  |  |
| 0 | 121,388 (77.0) | 7,840 (75.4) | <0.001 |
| 1 | 18,046 (11.4) | 1,169 (11.2) |  |
| 2 | 7,643 (4.8) | 486 (4.7) |  |
| 3 | 3,870 (2.5) | 268 (2.6) |  |
| >3 | 6,792 (4.3) | 633 (6.1) |  |
| Hospital inpatient visits |  |  |  |
| 0 | 130,220 (82.6) | 8,649 (83.2) | <0.001 |
| 1 | 18,013 (11.4) | 1,053 (10.1) |  |
| 2 | 6,112 (3.9) | 423 (4.1) |  |
| >2 | 3,394 (2.2) | 271 (2.6) |  |
| Had complex needs* |  |  |  |
| No | 127,997 (81.1) | 8,511 (81.9) | 0.07 |
| Yes | 29,742 (18.9) | 1,885 (18.1) |  |

* Either 1 physical condition plus depression or ≥2 physical conditions plus ≥1 mental health condition.
