# Supplementary material for: Geographic disparities in physical and mental health comorbidities and socioeconomic status of residence among Medicaid beneficiaries in Utah
Source: Front Public Health. 2025 Jan 6;12:1454783. doi: 10.3389/fpubh.2024.1454783 (PMC11743258; doi:10.3389/fpubh.2024.1454783)
Supplement: Supplementary file 2 [file Supplementary_file_1.docx]

Supplementary Figure. Definition of complex needs among adult Medicaid beneficiaries — Utah, 2017

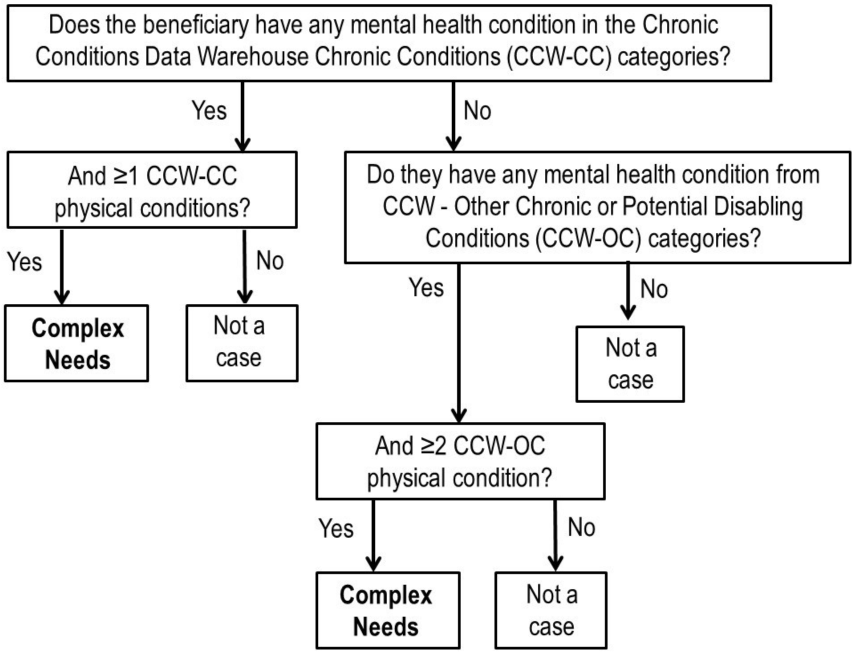

Chronic physical conditions: Acquired Hypothyroidism; Acute Myocardial Infarction; Alzheimer's Disease, Related Disorders, or Senile Dementia; Anaemia; Asthma; Atrial Fibrillation; Benign Prostatic; Hyperplasia; Breast Cancer; Colorectal Cancer; Endometrial Cancer; Lung Cancer; Prostate Cancer; Cataract; Chronic Kidney Disease; Chronic Obstructive Pulmonary Disease; Diabetes; Glaucoma; Heart Failure; Hip/Pelvic Fracture; Hyperlipidaemia; Hypertension; Ischemic Heart Disease; Osteoporosis; Rheumatoid Arthritis or Osteoarthritis; Stroke or Transient Ischemic Attack

Chronic mental health conditions: Attention-deficit/hyperactivity disorder Conduct Disorders and Hyperkinetic Syndrome; Alcohol Use Disorders; Anxiety Disorders; Autism Spectrum Disorders; Bipolar Disorder; Drug Use Disorders; Intellectual Disabilities and Related Conditions; Learning Disabilities and Other Developmental Delays; Personality Disorders; Post-Traumatic Stress Disorder; Schizophrenia and Other Psychotic Disorders; Traumatic Brain Injury and Nonpsychotic Mental Disorders Due to Brain Damage
